# *N*-Glycomic Signature of Stage II Colorectal Cancer and Its Association With the Tumor Microenvironment

**DOI:** 10.1074/mcp.RA120.002215

**Published:** 2021-02-11

**Authors:** Fanny Boyaval, René van Zeijl, Hans Dalebout, Stephanie Holst, Gabi van Pelt, Arantza Fariña-Sarasqueta, Wilma Mesker, Rob Tollenaar, Hans Morreau, Manfred Wuhrer, Bram Heijs

**Affiliations:** 1 Department of Pathology, Leiden University Medical Center, Leiden, the Netherlands; 2 Center for Proteomics & Metabolomics, Leiden University Medical Center, Leiden, the Netherlands; 3 Department of Surgery, Leiden University Medical Center, Leiden, the Netherlands; 4 Department of Pathology, Amsterdam University Medical Center, Amsterdam, the Netherlands

**Keywords:** colorectal cancer, *N*-glycosylation, mass spectrometry imaging, oligosaccharides, MALDI-MSI, molecular histology, ACN, acetonitrile, CRC, colorectal cancer, FFPE, formalin-fixed paraffin-embedded, LS, longer survival, MALDI-MSI, matrix-assisted laser desorption/ionization mass spectrometry imaging, mQ, milli-Q water, PC, principal component, ROI, regions of interest, *S/N*, signal-to-noise, SI, stroma interface, SS, shorter survival

## Abstract

The choice for adjuvant chemotherapy in stage II colorectal cancer is controversial as many patients are cured by surgery alone and it is difficult to identify patients with high risk of recurrence of the disease. There is a need for better stratification of this group of patients. Mass spectrometry imaging could identify patients at risk. We report here the *N*-glycosylation signatures of the different cell populations in a group of stage II colorectal cancer tissue samples. The cancer cells, compared with normal epithelial cells, have increased levels of sialylation and high-mannose glycans, as well as decreased levels of fucosylation and highly branched *N*-glycans. When looking at the interface between cancer and its microenvironment, it seems that the cancer *N*-glycosylation signature spreads into the surrounding stroma at the invasive front of the tumor. This finding was more outspoken in patients with a worse outcome within this sample group.

## Highlights

• *N*-glycan MS imaging as tool for better cancer stratification.
• Increased levels of sialylated and high-mannose glycans in CRC stage II cancer.
• Characterization of the interaction between cancer and its direct microenvironment.
• Insight into *N*-glycosylation signature of high-risk patients with short survival.

## Graphical Abstract

**Figure:**
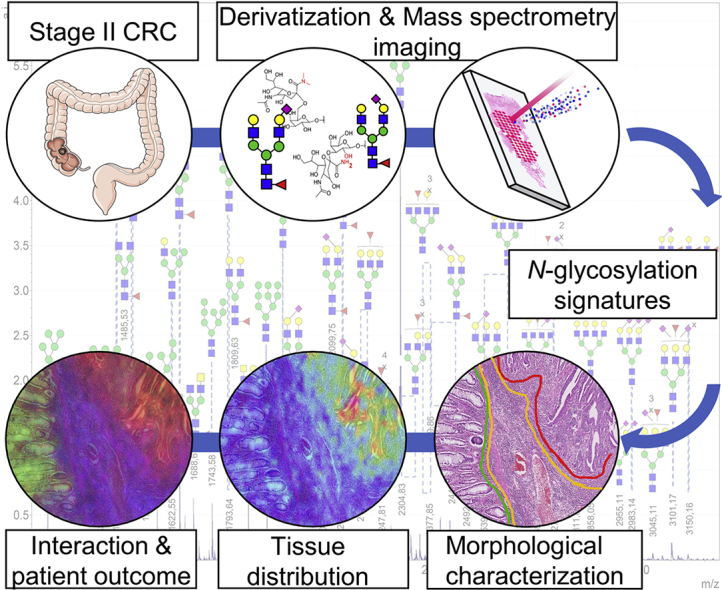

## In Brief

There is a need for better stratification of patients with stage II colorectal cancer as the treatment option remains controversial. Using MS imaging, we have characterized the *N*-glycosylation signatures of the different cell populations in stage II CRC tissues. We found, looking at the tumor microenvironment, that the cancer *N*-glycosylation signature spreads into the surrounding stroma at the invasive front of the tumor. This finding was more outspoken in patients with a worse outcome.

Colorectal cancer (CRC) is the third most lethal (8.2% of the total cancer-related deaths in 2018), and fourth most commonly diagnosed cancer worldwide (6.1% in 2018), and these numbers have continued to increase yearly (1, 2). Mortality has declined progressively in many Western countries due to population-based screening programs, resulting in higher detection and local treatment of precursor lesions (adenomas) and early adenocarcinomas. Patients with CRC are stratified using the eighth edition of the tumor-lymph node-metastasis staging system based on the depth of invasion of the primary tumor, nodal involvement, and presence of distant metastases (https://cancerstaging.org/Pages/default.aspx). Patients with stage I CRC have a 5-year survival rate of 98.5%, which decreases to an average of 75.7% and 56.7% for stage II and III patients, (3). According to the Dutch guideline for the treatment of colorectal cancer, stage II patients are not treated with adjuvant chemotherapy. However, 20% of these patients would probably benefit from it as they are likely to have recurrence of the disease after surgery. Identifying this group of patients is challenging. Despite the definition of high-risk stage II patients according to T-stage, the amount of lymph nodes sampled, and the presence of lymphovascular invasion, the effect of adjuvant chemotherapy was not evident in all high-risk patients and therefore remained controversial. Recently, a large effort was made to better stratify patients with CRC into biologically and clinically distinct subtypes using different factors, like histopathological features such as the tumor-stroma ratio (4, 5) or based on gene expression profiles with the consensus molecular subtype classification (6), or other expression-based methods such as the ColoPrint signature (Oncotype DX) (7, 8). However, none of these current classifiers allows for a clear and unambiguous stratification of these high-risk stage II patients. To get a clear understanding of the high-risk cases, and find molecular signatures that allow stratification of this patient group, a thorough molecular characterization of the different morphologies and cell populations within the stage II tumors is needed.

*N*-glycans, common oligosaccharidic post-translational protein modifications, play a major role in fundamental molecular and cellular processes occurring in all tissues and cancer tissues in particular. These include angiogenesis, immune modulation, metastasis formation, tumor cell invasion, cell signaling, and communication (9, 10). Alterations in glycosylation patterns have been associated with the development and progression of various types of cancer (11, 12). In CRC, these glycomic changes have shown to be discriminative between normal and cancerous epithelial cell lines and potentially reflect tumor stage specificity (13, 14, 15, 16, 17). However, it should be noted that the majority of studies on glycosylation changes in CRC were conducted using human serum samples or cell culture material. Studies conducted using CRC tissue samples were performed on homogenized tissue and therefore did not retain any morphological information to reflect molecular intratumor heterogeneity (13, 18).

To address this challenge, we have used matrix-assisted laser desorption/ionization mass spectrometry maging (MALDI-MSI), a spatially resolved molecular profiling strategy that can be directly correlated to histopathological information, allowing us to study the molecular landscape underlying the intratumor heterogeneity (19, 20). Powers *et al.* (21) pioneered *N*-glycan MALDI-MSI to study *N*-glycans directly from tissue sections. More recently, Holst *et al.* (22) developed an on-tissue linkage-specific sialic acid derivatization method to enable the spatially correlated characterization of differently linked sialic acids in formalin-fixed paraffin-embedded (FFPE) tissues. Native, nonderivatized, *N*-glycan MALDI-MSI has been successfully applied to a variety of different human tumor and tissue cohorts, including hepatocellular carcinoma and ovarian, prostate, myxoid liposarcoma, and pancreatic cancer (23, 24, 25, 26). Here we describe, for the first time, results of *N*-glycan MALDI-MSI after linkage-specific sialic acid derivatization on a group of patients with stage II CRC, with the aim of identifying the glycomic intratumor heterogeneity and provide new molecular insights that could benefit accurate patient stratification.

## Experimental Procedures

### Chemicals and Reagents

Ethanol was obtained from Merck. Dimethylamine, α-cyano-4-hydroxycinnamic acid, dimethylsulfoxide, 1-hydroxybenzotriazole hydrate, sodium hydroxide, and TFA were obtained from Sigma-Aldrich. HPLC SupraGradient acetonitrile (ACN) was obtained from Biosolve. 1-Ethyl-3-(3-dimethylaminopropyl) carbodiimide from Fluorochem. Recombinant peptide *N*-acetyl-beta-glucosaminyl asparagine amidase PRIME-LY Glycosidase from *Flavobacterium meningosepticum* was purchased from N-Zyme Scientifics. The peptide calibration standard was purchased from Bruker Daltonics. All buffers were prepared using Milli-Q water (mQ) generated from a Q-card 2 system (Millipore).

### Tissue Sample Collection

Tissues from colorectal cancer resections (n = 16) used for this study were obtained from the department of pathology from Leiden University Medical Center. Tissues were FFPE according to routine protocols of the department. H&E-stained sections were annotated by two experienced gastrointestinal pathologists (H. M. and A. F. -S.). Annotations included “cancer” (C) corresponding to the malignant epithelial cells, “adjacent normal colon epithelium” (N), “distant stroma”, for the reactive stroma surrounding the cancer areas, and “stroma interface” (SI) for the stroma region directly connected to the invasive front of the cancer. All samples were handled in a coded fashion, according to the national ethical guidelines (“Code for Proper Secondary Use of Human Tissue”, Dutch Federation of Medical Scientific Societies).

### Sample Preparation for MALDI-MSI Analysis

FFPE tissue blocks were sectioned with a microtome Leica (Leica Biosystems RM2245 Microtome) at 6-μm thickness. Tissues sections were mounted in pairs onto poly-L-lysine- and indium tin oxide-coated glass slides (Bruker Daltonics). Before tissue mounting, glass slides were cleaned in 70% ethanol for 10 min and coated with a 0.05% poly-L-lysine solution in mQ. All sections were dried overnight at 37 °C, stored at 4 °C, and then prepared following the procedure by Holst *et al.* (22). In brief, paraffin was removed by heating the slides for 1 h at 65 °C followed by two consecutive washes in xylene (10 min and 5 min, respectively). Tissues were rehydrated in ethanol baths (100% ethanol, twice for 2 min), followed by water baths (twice for 5 min), and dried for 10 min in a vacuum desiccator. On-tissue derivatization was performed by incubating the tissues slides in derivatization solution (250 mM 1-ethyl-3-(3-dimethylaminopropyl) carbodiimide, 500 mM 1-hydroxybenzotriazole hydrate, and 250 mM dimethylamine in dimethylsulfoxide) for 1 h at 60 °C, followed by addition of a 25% ammonia solution (1:0.4 v/v derivatization solution-ammonia) and further incubation for 2 h at 60 °C, both protected from evaporation. After derivatization, tissue sections were rinsed thoroughly with 100% ethanol followed by sequential washes in 100% ethanol (2 × 2 min) and water (2 × 5 min). Slides were dried in a vacuum desiccator (10 min). On-tissue digestion was performed applying 10 layers at 10 μl/min of peptide *N*-acetyl-beta-glucosaminyl asparagine amidase (0.1 μg/μl in Tris buffer; from N-Zyme Scientifics) using a SunCollect sprayer (SunChrom). *N*-glycans were released overnight at 37 °C in a humid environment. After incubation, slides were dried in a vacuum desiccator for 10 min, followed by matrix application (5 mg/ml α-Cyano-4-hydroxycinnamic acid in 50:49.9/0.1 (%v/v) ACN:mQ:TFA) using the SunCollect sprayer (6 layers at (1) 10 μl/min, (2) 20 μl/min, (3) 30 μl/min, (4+) 40 μl/min).

### MALDI-MSI Analysis

*N*-glycan MALDI-MSI was performed in positive-ion reflectron mode on a rapifleX MALDI-TOF/TOF-MS instrument (Bruker Daltonics) using a *m/z* range of 900 to 3300 Th, 1000 laser shots per pixel, 50 × 50 μm^2^ pixel size. MSI data acquisition was enabled by the flexImaging software (flexImaging 4.0 Build 32, Bruker Daltonics). After the MSI analysis, excess MALDI-matrix was removed by washing twice in 70% ethanol (5 min each), and tissues were stained with H&E after routine histopathological procedures.

High-resolution microscopy images of the stained sections were taken using a digital slide scanner (IntelliSite Pathology Ultra-Fast Scanner, Philips) and coregistered with the MALDI-MSI data in the flexImaging software. For each tissue, regions of interest (ROIs) were drawn following the annotations of expert pathologists. Spectra, ROIs, and coregistered H&E images were imported into SCiLS Lab software 2016b (Version 4.01.8781, Bruker Daltonics).

### *N*-Glycan Extraction and Identification

For identification by MS/MS analysis, consecutive tissue sections were cut and mounted on Starfrost adhesive microscope glass slides and then deparaffinized and rehydrated according to the previous protocol describing the sample preparation for MALDI-MSI analysis. Released *N*-glycans were then extracted and derivatized according to previously described methods (22). Dried samples were cocrystallized with sDHB matrix (5 mg/ml in 50% ACN, 1 mM NaOH). Spectra were recorded using the rapifleX mass spectrometer, and tandem MS/MS was performed for glycan identification.

### Data Preprocessing and Analysis

The total ion current-normalized overall average spectrum of the full dataset was exported to .csv-format from SCiLS Lab and loaded in the open-source software mMass (http://www.mmass.org (27)). The average spectrum was processed in mMass using the following parameters: baseline subtraction with 15 precision and 25 relative offset; smoothing with Savitzky-Golay smoothing, window size: 0.05 *m/z* and four cycles; internally recalibration Peak picking was performed with a signal-to-noise (*S/N*) threshold of 3 (*S*/*N* ≥3) followed by deisotoping (maximum charge: 1, isotope mass tolerance: 0.15 *m/z*, isotope intensity tolerance: 50%). *N*-glycan compositions were assigned using the Glyco-Peakfinder tool in GlycoWorkbench (version 2.1 stable build 146, http://www.eurocarbdb.org/) as well as MS/MS data (supplemental Data2) (28). The assigned *N*-glycan compositions were used in the “composition list” for MassyTools, a data processing tool for targeted high-throughput *N*-glycan MALDI-MS data extraction (29). In MassyTools, an internal calibration based on a list of internal calibrants was performed. Spectra with at least four calibrants were kept for further data processing. The data quality of the areas was checked by keeping only the ROIs that have 50% of the total intensity with a *S/N* lower than nine. Furthermore, analytes were included for further analysis if the majority of the ROI spectra (≥50%) had a mass error below 20 ppm, an *S/N* ≥9, and an isotopic pattern quality score ≤0.5. Multiple ROIs coming from the same patient and with the same morphology were averaged, and the relative intensity of all *N*-glycan was rescaled to 100%. Derived traits were calculated from single *N*-glycans using an in-house R script (http://www.rstudio.com, supplemental Data1 S3). Statistical analyses were achieved in MATLAB (R2016a, 9.0.0.341360) by first applying a feature selection using the Kruskal-Wallis test and a nonparametric one-way ANOVA. Then, *N*-glycans with a *p*-value <0.05 cutoff were tested for significance using a nonparametric Wilcoxon rank-sum test, followed by Benjamini-Hochberg multiple testing correction. The principal component analysis test was performed using SIMCA (version 13.0.3.0 by UMETRICS).

### Experimental Design and Statistical Rationale

In this study, the *N*-glycome from 16 FFPE tissues from patients with stage II CRC were analyzed by MALDI-MSI. Owing to limited sample availability, technical replicates were performed for four cases. Owing to sample preparation, the samples were analyzed by pair, one longer survival (LS) patient and one shorter survival (SS) patient tissue together. The pairs and the technical replicates were analyzed in a random order. For each patient group, at least 6 biological replicates were included. The technical replicates show similar spectra and *N*-glycan distributions as shown in Supplemental Fig. S3, indicating the reproducibility of the applied method. No samples were excluded from the analysis. The Kruskal-Wallis test, Wilcoxon rank-sum test, and multiple testing correction were used in this study.

## Results

**Fig. 1:**
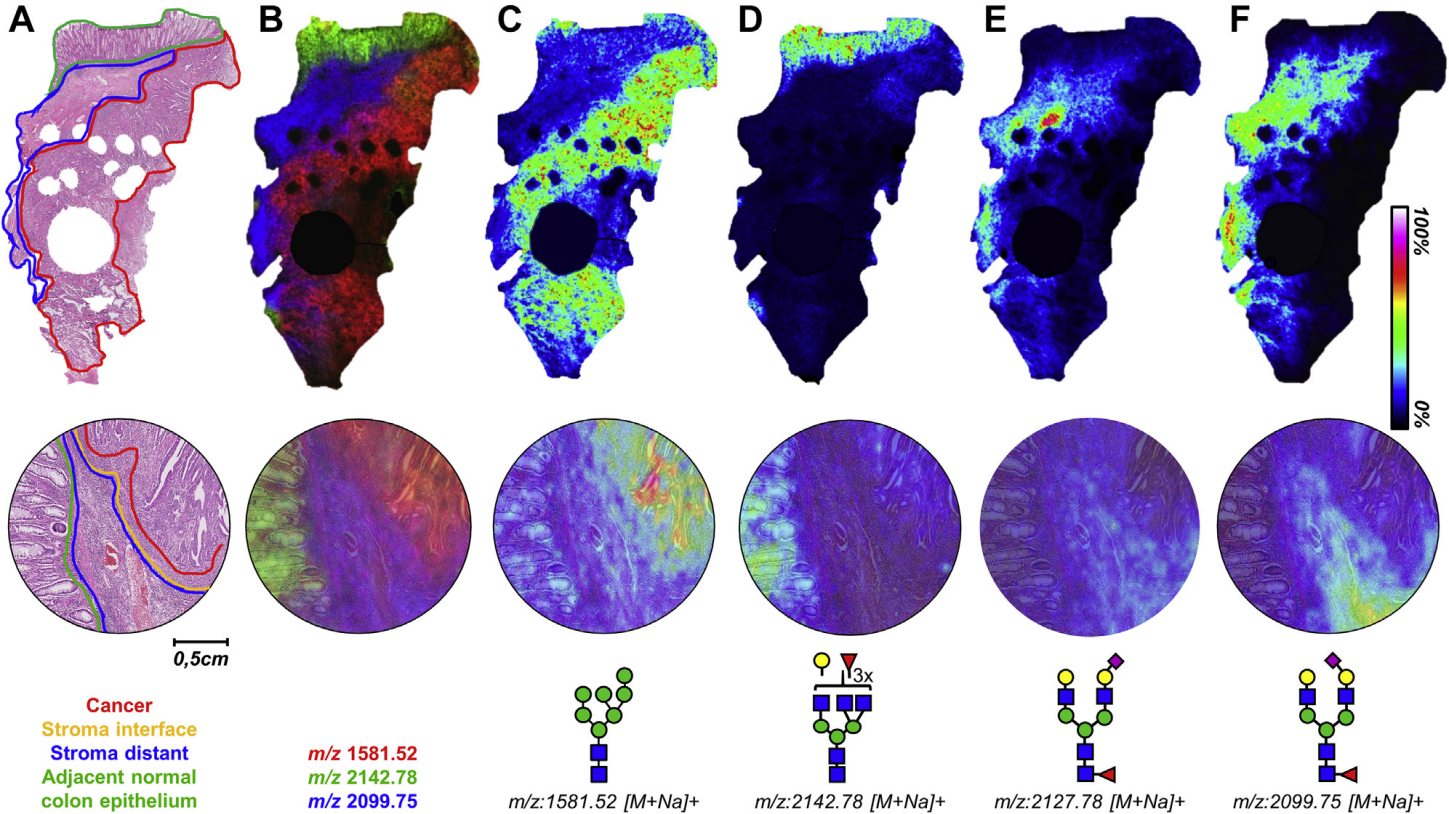
**Morphology-specific *N*-glycosylation signatures.***A*, H&E-stained stage II CRC tissue (2× magnification) with the corresponding morphological annotations. The *bottom panel* shows a 20× magnification of an area of the tissue annotated. *B*, the overlay of *m/z* 1581.52*,* Hex_7_HexNAc_2_ (*red*); *m/z* 2142.78, Hex_4_dHex_3_HexNAc_5_ (*green*); and *m/z* 2099.75, Hex_5_dHex_1_HexNAc_4_NeuAc(α2,3)_1_ (*blue*). *C*–*F*, visualizations of the distribution of four different *N*-glycans throughout the same tissue and zoom in one area with their corresponding structures. Images were obtained from a TIC-normalized dataset. The *round gaps* (*large* and *small*) inside the tissue are the result of tissue sampling for diagnostic purposes. NeuAc(α2,3), *α*2,3-linked N-acetylneuraminic acid; CRC, colorectal cancer; dHex, fucose; Hex, hexose; HexNAc, N-acetylhexosamine.

In this study, FFPE tissues from 16 patients with stage II CRC were analyzed by *N*-glycan MALDI-MSI. Patient characteristics are presented in Table 1. After *N*-glycan MALDI-MSI analysis and H&E staining, specific morphological areas were annotated, including “cancer” (C), “adjacent normal colon epithelium” (N), “distant stroma”, and SI (“Tissue Sample Collection” in Experimental Procedures and Fig. 1*A*). A total of 108 glycan compositions (supplemental Data1 S1 and S2) were detected and analyzed. supplemental Fig. S1 shows the overall average spectrum of the cohort with the corresponding *N*-glycan masses. These glycans were used to calculate derived traits based on the biosynthetic assembly of the *N*-glycans. The derived traits include, for example, overall *N*-glycan-type, the number of antennae, and the presence of fucose, and/or (linkage specific) sialylation (supplemental Data1 S3).

**Table 1:** Characteristics of patient cohort

Patients	16
Median age (IQR)	72 (9)
Gender	
Male (%)	6 (37.5)
Female (%)	10 (62.5)
Pathologic classification	
pT3N0M0	16
Differentiation grade	
Well	8
Medium	0
Poor	8
Microsatellite instability	
Stable	8
Instable	6
Unknown	2
Tumor-stroma ratio	
Stroma low	4
Stroma high	12
Mean OS (months)	114 (SD: 73)
Median 5-year survival (months)	123 (IQR: 120)
Recurrence (No. of patients)	6

IQR, interquartile range; MSS, microsatellite stable; OS, overall survival.

### Morphology-Specific *N*-Glycosylation Signatures

To evaluate whether the different morphologies were distinguishable by their *N*-glycan profiles, a principal component analysis was performed on the relative abundances of all *N*-glycans. This resulted in a model with five principal components (PCs) explaining 69% of the total variation. PC1 and PC2, explaining 24% and 16% of the variation, respectively, showed a clear separation between cancer, stroma, and adjacent normal colon epithelium, indicating that the different morphologies have distinct *N*-glycomic profiles (supplemental Fig. S2).

In Figure 1, several of the glycan are visualized, and various differences in their distribution can be observed. For example, the high-mannose-type *N*-glycan Hex_7_HexNAc_2_ (*m/z* = 1581.5 [M+Na]^+^, where Hex = hexose, and HexNAc = *N*-acetylhexosamine) is predominantly detected in the cancer areas (Fig. 1*C*), while trifucosylated complex-type *N*-glycan Hex_4_dHex_3_HexNAc_5_ (*m/z* = 2142.78 [M+Na]^+^, dHex = fucose) is detected predominantly in the normal adjacent epithelium areas (Fig. 1*D*, supplemental Fig. S2). Heterogeneity in the cancer areas was studied by comparing the glycomic signatures of the invasive front (cancer-side of the interface) and the bulk of the cancer area (distant from the interface), although no glycosylation differences appeared (data not shown).

The chemical derivatization strategy applied in this study modified sialic acids, which resulted in a mass shift, allowing for the *in situ* distinction between α2,3-linked *N*-acetylneuraminic acid (NeuAc(α2,3)) and α2,6-linked *N*-acetylneuraminic acid (NeuAc(α2,6)) residues as shown in Figure 1, *E* and *F* (22). For example, the sialylated, diantennary complex-type *N*-glycan Hex_5_dHex_1_HexNAc_4_NeuAc_1_ (NeuAc = *N*-acetylneuraminic acid) was detected having different sialic acid configurations. *N*-glycan Hex_5_dHex_1_HexNAc_4_NeuAc(α2,6)_1_ was found to be distributed throughout the stroma but with lower intensity in the areas directly surrounding the tumor and not in the tumor (Fig. 1*E*). In contrast, Hex_5_dHex_1_HexNAc_4_NeuAc(α2,3)_1_ was highly expressed in stroma, with higher intensities in the interface areas than the rest of the stroma and in the invasive front of the tumor (Fig. 1*F*, supplemental Fig. S2). These observations highlight the importance of distinguishing the different sialic acid linkages due to the molecular heterogeneity of the tumor tissue.

#### Changes in Sialylated, Fucosylated, and High-Mannose N-Glycans in Cancer

*N*-glycan profiles of the cancer and adjacent normal epithelial areas were first compared as CRC is a carcinoma, that is, a cancer developing from epithelial cells. A Wilcoxon rank-sum test followed by a Benjamini-Hochberg multiple testing correction was used to compare derived *N*-glycan traits between the two groups (*p*-value ≤0.05) (supplemental Data1 S4 and S5). A large number of the derived traits (*n* = 99) showed significant differences between the two groups, a few of those are further highlighted below.

#### Sialylated and Fucosylated Complex-Type N-Glycans

Overall complex-type *N*-glycans with sialylation (FC_*C/N*_ = 1.31, *p*-value = 0.002) were more abundant in cancer areas than the normal adjacent epithelium, even if not all individual *N*-glycans followed the same trend. When separating complex-type *N*-glycans with sialylation into the differently linked sialic acids, it appeared that α2,6-linked sialylation (FC_*C/N*_ = 1.55, *p*-value = 0.001) varied substantially more between cancer and adjacent normal epithelium than α2,3-linked sialic acids (FC_*C/N*_ = 1.17, *p*-value = 0.039). Not only did the overall level of sialylation increase in cancer areas, the sialylation per galactose also increased for the diantennary (A2GS; FC_*C/N*_ = 1.17, *p*-value = 0.002), triantennary (A3GS; FC_*C/N*_ = 1.38, *p*-value = 0.001), and tetra-antennary (A4GS; FC_*C/N*_ = 1.27, *p*-value = 0.018) complex-type glycans.

**Fig. 2:**
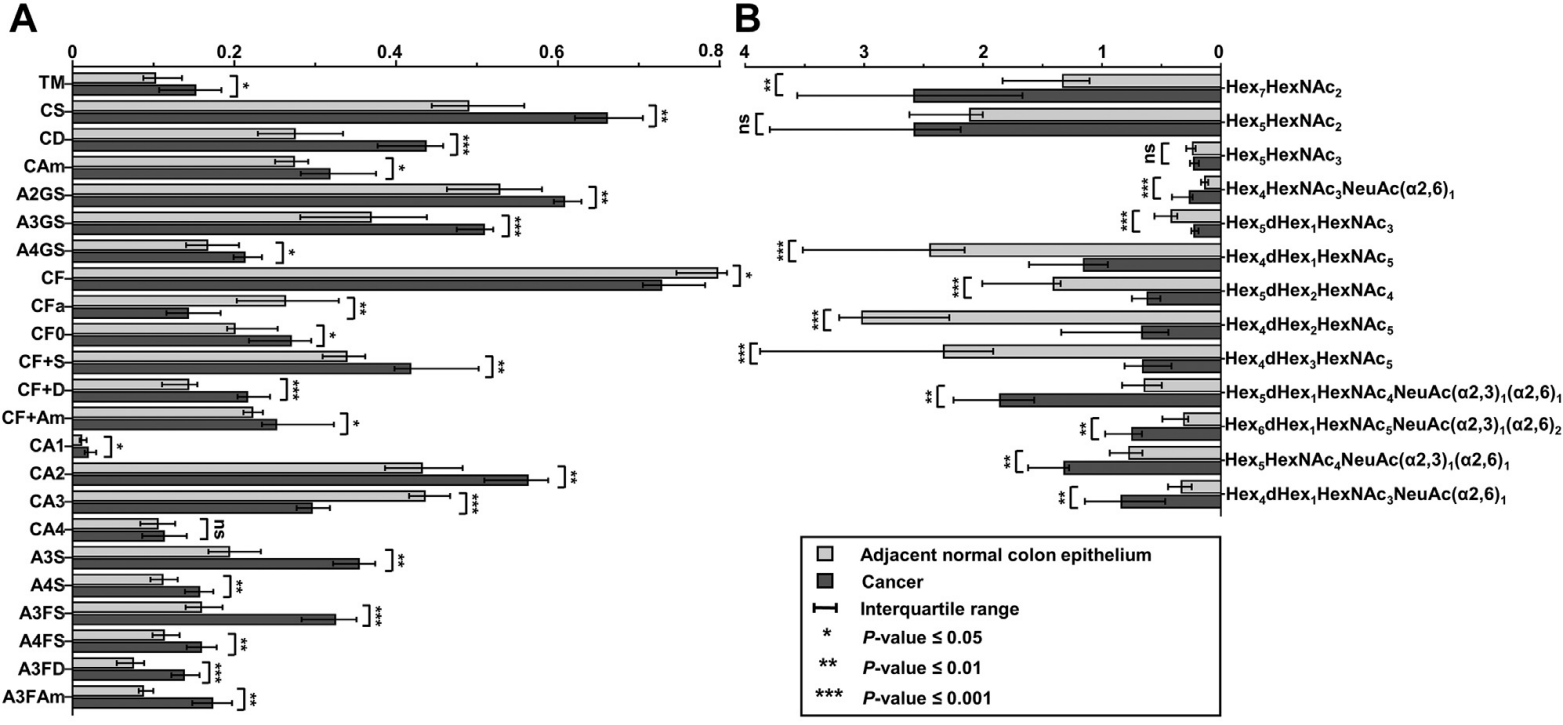
**Glycosylation differences between cancer and adjacent normal colon epithelium.***A*, the median relative abundances of derived *N*-glycan traits and *B*, single *N*-glycans were found to be significantly altered between cancer and normal colon epithelia. NeuAc(α2,3), α2,3-linked N-acetylneuraminic acid; NeuAc(α2,6), α2,6-linked N-acetylneuraminic acid; Hex, hexose; dHex, fucose; HexNAc, N-acetylhexosamine (refer supplemental Data1 S3 for trait abbreviations).

In contrast, the total abundance of fucosylated complex-type *N*-glycans (CF; FC_C/N_ = 0.94, *p*-value = 0.024), as well as the abundance of complex-type with multiple fucosylations (FC_C/N_ = 0.60, *p*-value = 0.006), decreased in cancer. Consequently, we observed an increase of complex-type *N*-glycans without fucosylation (CF0; FC_C/N_ = 1.24, *p*-value = 0.024). The *N*-glycans carrying one or more fucoses, Hex_5_dHex_1_HexNAc_3_ (FC_C/N_ = 0.46, *p*-value = 0.001), Hex_4_dHex_1_HexNAc_5_ (FC_C/N_ = 0.45, *p*-value = 0.001), Hex_5_dHex_2_HexNAc_4_ (FC_C/N_ = 0.38, *p*-value = 0.001), and Hex_4_dHex_3_HexNAc_5_ (FC_C/N_ = 0.23, *p*-value = 0.001) were vastly lower in cancer as shown in Figure 2 and supplemental Data1 S4. Of note, high expression of Hex_4_dHex_3_HexNAc_5_ was specific for adjacent normal colon epithelium (Fig. 1*D*).

Although overall fucosylation was decreased in cancer, complex-type *N*-glycans with both fucosylation and sialylation were more abundant in cancer (CF+S; FC_*C/N*_ = 1.31, *p*-value = 0.003), which held true for both of the sialic acid linkages (CF+D; FC_*C/N*_ = 1.69, *p*-value = 0.001; and CF + Am; FC_*C/N*_ = 1.2, *p*-value = 0.008). When separating the complex-type species based on their number of antennae, in combination with both sialylation and fucosylation, we observed tetra-antennary glycans such as Hex_7_dHex_3_HexNAc_6_NeuAc(α2,6)_1_ (FC_*C/N*_ = 4.13, *p*-value = 0.008) and Hex_7_dHex_1_HexNAc_6_NeuAc(α2,3)_1_(α2,6)_1_ (FC_*C/N*_ = 3.29, *p*-value = 0.002) and triantennary glycans such as Hex_6_dHex_1_HexNAc_5_NeuAc(α2,3)_1_(α2,6)_1_ (FC_*C/N*_ = 2.27, *p*-value = 0.002) or Hex_6_dHex_1_HexNAc_5_NeuAc(α2,3)_1_(α2,6)_2_ (FC_*C/N*_ = 2.14, *p*-value = 0.001) to be strongly elevated in the cancerous area.

#### Antennarity of Complex-Type N-Glycans

Diantennary and triantennary glycans were the most abundant complex-type glycans throughout the tissues, whereas the abundances of monoantennary and tetraantennary were much lower. Between cancer and adjacent normal epithelial areas, increased abundance of diantennary glycans (CA2; FC_*C/N*_ = 1.26, *p*-value = 0.009) and a concomitant decrease in triantennary glycans (CA3; FC_*C/N*_ = 0.67, *p*-value = 0.001) was observed in cancer. Although at a low overall intensity, monoantennary type increased (CA1; FC_*C/N*_ = 1.86, *p*-value = 0.026) in cancer. Tetraantennary glycans were not changing significantly.

#### High-Mannose N-Glycans

Visualization of the individual high-mannose *N*-glycan distributions (Hex_5_HexNAc_2_, Hex_6_HexNAc_2_, Hex_7_HexNAc_2_, Hex_8_HexNAc_2_, and Hex_9_HexNAc_2_) showed that they appeared to be more present in the cancer areas. The corresponding derived trait was at the trend-level significance (total high mannose; fold change FC_*C/N*_ = 1.48, *p*-value = 0.051, supplemental Data1 S5). The only significant high-mannose glycan between cancer and adjacent normal epithelium was Hex_7_HexNAc_2_ (FC_*C/N*_ = 1.95, *p*-value = 0.009), and its distribution correlated directly with the cancer area as shown in Figure 1*C*.

### Characterization of the Tumor Microenvironment

**Fig. 3:**
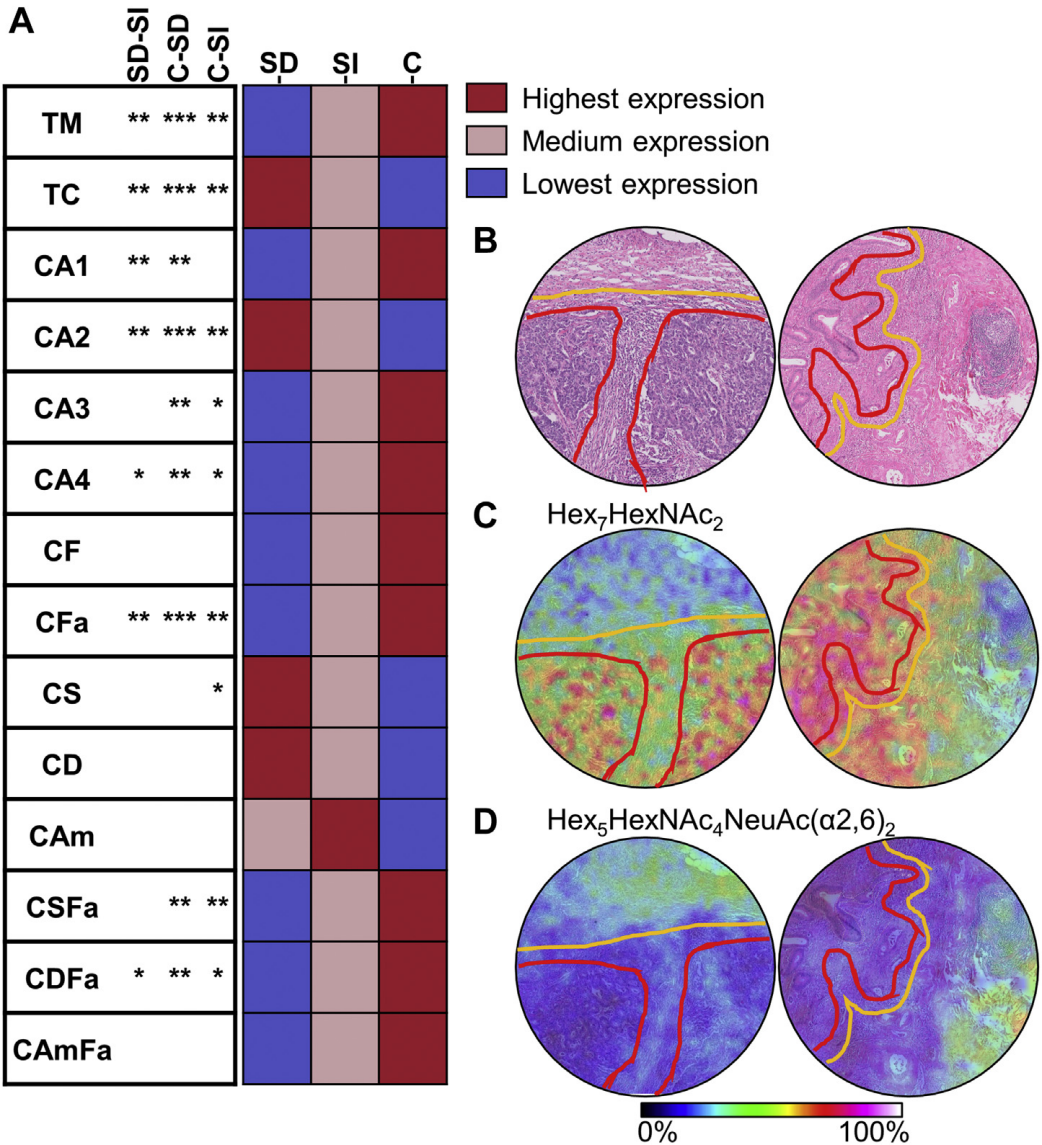
**Glycosylation differences between cancer and its microenvironment.***A*, on the *left*, the comparison of the derived traits between the different morphologies; SD *versus* SI, C *versus* SD, and C *versus* SI. ∗*p*-value ≤0.05, ∗∗*p*-value ≤0.01, ∗∗∗*p*-value ≤0.001, blank is not significant. On the *right*, the heat map of the relative abundance of the derived traits per morphologies with the corresponding color scale. *B*, H&E staining with the morphological annotations of tissues. *C* and *D*, visualization of the distribution of two single *N*-glycans with the morphological annotations. C, cancer; NeuAc(α2,6), α2,6-linked N-acetylneuraminic acid; Hex, hexose; HexNAc, N-acetylhexosamine; SD, stroma distant; SI, stroma interface (refer supplemental Data1 S3 for traits abbreviations).

To determine the glycomic heterogeneity of the tumor microenvironment, we annotated distant stroma and interface stroma areas (Fig. 1*A*) and compared their molecular profiles. A total of 41 *N*-glycans and 26 derived traits differed between the two groups (supplemental Data1 S6 and S7). Distant stroma was characterized by a low abundance of high-mannose glycans (total high mannose; FC_SI/SD_ = 0.50, *p*-value = 0.004), multifucosylated complex *N*-glycans (complex-type with multiple fucosylations; FC_SI/SD_ = 0.66, *p*-value = 0.009), and a high abundance of complex-type glycans (total complex type; FC_SI/SD_ = 1.07, *p*-value = 0.003), in particular diantennary complex-type glycans (CA2; FC_SI/SD_ = 1.12, *p*-value = 0.009). Sialylation was similar for the two groups, although a decrease in sialylation combined with multiple fucosylations was observed in distant stroma (CSFa; FC_SI/SD_ = 0.59, *p*-value = 0.011), especially multiple fucosylations in combination with α2,6-linked sialic acid (CDFa; FC_SI/SD_ = 0.48, *p*-value = 0.014). When comparing the differences in glycosylation between distant stroma, interface stroma, and cancer areas, it appeared that the interface stroma followed a similar glycosylation trend to the cancer areas (Fig. 3, supplemental Data1 S8 and S9). This was especially true when comparing the abundances of high-mannose-type glycans, complex-type glycans, their antennarity, and fucosylation. The high-mannose-type glycan Hex_7_HexNAc_2_ shows clear presence in the cancer area, with gradually lower expression in the interface and the stroma lining the tumor (Fig. 3*C*). The complex-type glycan Hex_5_HexNAc_4_NeuAc(α2,6)_2_ was very abundant in the distant stroma, lower in the interface stroma, and hardly detected in the cancer area (Fig. 3*D*).

### Prognostic Value of *N*-Glycosylation Signatures

When comparing groups according to the overall survival, we did not find differences in the cancer areas between two survival groups: LS > 5-years, and SS < 5-years survival (supplemental Data1 S11). However, we did find differences in the interface stroma within the two groups. The interface stroma of the LS group was characterized by an increased galactosylation per antenna in diantennary complex-type glycans compared with the SS group (A2G; FC_SI-LG/SS_ = 1.07, *p*-value = 0.045; supplemental Data1 S10). This increase in galactosylation was also confirmed by other traits; it also appeared in diantennary glycans with and without fucosylation, as well as those without sialylation (A2FG; FC_SI-LG/SS_ = 1.10, *p*-value = 0.045; A2F0G; FC_SI-LG/SS_ = 1.01, *p*-value = 0.033; A2S0G; FC_SI-LG/SS_ = 1.20, *p*-value = 0.045; supplemental Data1 S10).

## Discussion

In the present study, MALDI-MSI was applied to evaluate the heterogeneity of *N*-glycosylation in the stage II CRC and its microenvironment to stratify patients with different outcomes.

We first investigated the differences in *N*-glycosylation between normal adjacent epithelial cells and cancer cells, where we observed an increase in sialylated *N*-glycans in cancer areas. Especially so the *N*-glycans with α2,6-linked sialic acids, which we were able to discern through the application of the linkage-specific sialic acid derivatization. It is important to note that the level of sialylation increased independently of the number of galactose residues, indicating that its increase was not merely an effect of increased antennarity but likely due to the increased activity or presence of the sialyltransferases. These findings are in line with the current literature, as sialylation is known to play an important role in cellular recognition, cell adhesion, and cell signaling (30, 31, 32). Increased sialylation (both α2,3- and α2,6-linked) has been previously associated with different cancer types, including colon, stomach, and ovarian cancer (9, 30). Overexpression of β-galactoside α2,6sialyltransferase, the enzyme attaching α2,6-linked sialic acid to the lactosaminic termini of glycoproteins, has been associated with CRC tumorigenesis, metastasis, and protection against apoptosis (33, 34, 35, 36, 37). In the same line, expression of α2,3-linked sialylated structures was shown to be associated with metastatic potential in colorectal and gastric cancers (36, 38).

Our study revealed a decrease in overall fucosylation in cancer compared with the normal epithelium. The current literature is quite divided on the association and role of fucosylation in cancer. Several publications describe decreased fucosylation in both serum and tissues samples of patients with CRC (39, 40). Yet, other studies show increased fucosylation in cancer and have associated it to the metastatic potential of CRC (14, 41). Furthermore, due to the experimental setup, we were not able to distinguish between core and antenna fucosylation *in situ*. A recent study by West *et al.*, (42) published during the writing of this article, proved its feasibility with MALDI-MSI. Further investigation will be needed to address this issue.

In contrast to what is known from other cancer types, such as hepatocellular carcinoma and breast and pancreatic cancer, the abundance of triantennary complex-type glycan structures was decreased in cancer areas (26, 43, 44). Triantennary glycans, product of the *N*-acetylglucosaminyltransferases IV and V, are known to act against cell-cell and cell-matrix adhesion and therefore promote EMT and cell migration (45, 46, 47), correlating in turn with invasion and probably with disease stage (48, 49, 50). We studied stage II CRC, which is considered relatively early stage, and therefore, one could speculate that the increase in triantennary glycans could take place in more advanced stages and is therefore not apparent in this sample set.

Although at trend-level significance, the group of high-mannose-type *N*-glycans, early-phase intermediates of the biosynthetic maturation of *N*-glycans, was more abundant in cancer areas. Previous studies have shown increased levels of high-mannose-type *N*-glycans in several diseases, including CRC and breast cancer (17, 51, 52). However, the reason for the elevation of high-mannose-type *N*-glycan structures remains uncertain. It has been suggested that their increase in cancer is a consequence of precursor accumulation because of incomplete or limited *N*-glycan maturation (52).

The next step of our study was to compare the *N*-glycosylation signatures of the “distant stroma” and of the SI to the cancer areas signature. Tumors are complex systems, characterized by finely balanced and reciprocal interactions between the cancer cells and their direct microenvironment (53, 54). The tumor microenvironment, or stroma, consists predominantly of immune cells, extracellular matrix, and cancer-associated fibroblasts (55). The latter are known to have a high impact on cancer progression and remodeling of the extracellular matrix by secreting growth factors promoting tumor growth and induce a mesenchymal phenotype in cancer cells (4, 56, 57). Moreover, it has been shown that increase of cancer-associated fibroblast markers (PDGFR-β, FAP, CD45, CD31) in the invasive front of the cancer might contribute to the invasive behavior of CRC cells (4). From our results, we see that the invasive front (the cancer-side of the interface) and the bulk of the cancer areas (distant from the interface) did not show different glycosylation signatures. On the contrary, we did observe differences between interface stroma and distant stroma. We observed an increase in high-mannose glycans, sialylation of complex-type glycans, and an overall decrease in complex-type glycans in the interface stroma. It appears that the *N*-glycosylation signatures of the interface stroma is more similar to that of the cancer area than the distant stroma. As stated before, the intracancer glycosylation appeared homogeneous. Therefore, the differences between interface stroma and distant stroma can most likely be attributed to cancer-derived factors secreted toward the stroma, rather than stroma-derived factors secreted into the invasive front of the cancer. Furthermore, it appeared that the changes of the interface stroma glycosylation were stronger in the LS group. The glycosylation of the cancer did not vary between the groups according to survival, which suggests that the more aggressive cancers are interacting more strongly with their direct microenvironment.

The interface stroma of the group with LS was characterized by an increase in galactosylation per antenna in the diantennary complex-type glycans. Galactosylation was found to be decreased in lung cancer tissues and on the tumor-associated human carcinoma antigen in CRC (15, 58). Zhao *et al.* (15) hypothesized that the downregulated galactosylation on tumor-associated human carcinoma antigen in tumors may be correlated with CRC immune response. The stroma is largely orchestrated by inflammatory and immune cells, so the increased level of galactosylation in our study may be correlated to the inflammatory/immune response against cancer cells. This inflammatory reaction is stronger at the invasive front of the tumor and mainly in the group with LS.

In conclusion, the present study characterized the glycosylation signatures of different specific morphological features within CRC stage II tumors. As a result, we were able to directly compare cancer cells with adjacent normal colon epithelium and the cancer microenvironment. The interactions between cancer and stroma show that the cancer glycosylation signature is spreading into the adjacent stroma at the interface and that this interaction may play a role in survival of patients with stage II CRC. Although subject to validation of our findings in independent, larger CRC cohorts, our results affirm that *N*-glycan MALDI-MSI is useful for tissue profiling with potential applications in molecular pathology. In the future, these glycan changes can be exploited for a better stratification of patients with stage II CRC.

## Data Availability

All processed data compared between groups are available in supplemental Data1 and the curated data after MassyTools extraction and the annotated MS/MS data. MS/MS raw data can be found in supplemental Data2. The MS imaging data and the MS/MS raw spectrum have been deposited to the ProteomeXchange Consortium *via* the PRIDE (59) partner repository with the dataset identifier PXD021275 and the dataset identifier PXD021682, respectively. Further information can be requested to the lead contact, Bram Heijs (b.p.a.m.heijs@lumc.nl).

## Supplemental data

This article contains supplemental data.

## Conflict of interest

The authors declare no (financial) competing interests.

## Supplemental Data

Supplemental Figures S1–S3

Supplemental Data1 S1–S11

Supplemental Data2
