# Supplementary material for: N-Glycomic Signature of Stage II Colorectal Cancer and Its Association With the Tumor Microenvironment
Source: Mol Cell Proteomics. 2021 Feb 11;20:100057. doi: 10.1074/mcp.RA120.002215 (PMC7973300; doi:10.1074/mcp.RA120.002215)
Supplement: Supplemental Figures S1–S3 [file mmc1.docx]

**Supplementary information**

***N*-glycomic signature of stage II colorectal cancer and its association with the tumor microenvironment**

Fanny Boyaval^1,2^, René Van Zeijl^2^, Hans Dalebout^2^, Stephanie Holst^2^, Gabi van Pelt^3^, Arantza Fariña-Sarasqueta^1,4^, Wilma Mesker^3^, Rob Tollenaar^3^, Hans Morreau^1^, Manfred Wuhrer^2^, Bram Heijs^2*^

^1^ Department of Pathology, Leiden University Medical Center, Leiden, The Netherlands

^2^ Center for Proteomics & Metabolomics, Leiden University Medical Center, Leiden, The Netherlands

^3^ Department of Surgery, Leiden University Medical Center, Leiden, The Netherlands

^4^ Department of Pathology, Amsterdam University Medical Center, Amsterdam, The Netherlands

* Corresponding author: b.p.a.m.heijs@lumc.nl

Table of content:

**Supplementary Figure S1: MALDI-TOF-MS N-glycome spectra of stage II CRC Supplementary Figure S2: Separation of CRC morphologies based on their *N*-glycome Supplementary Figure S3: Technical replicates**


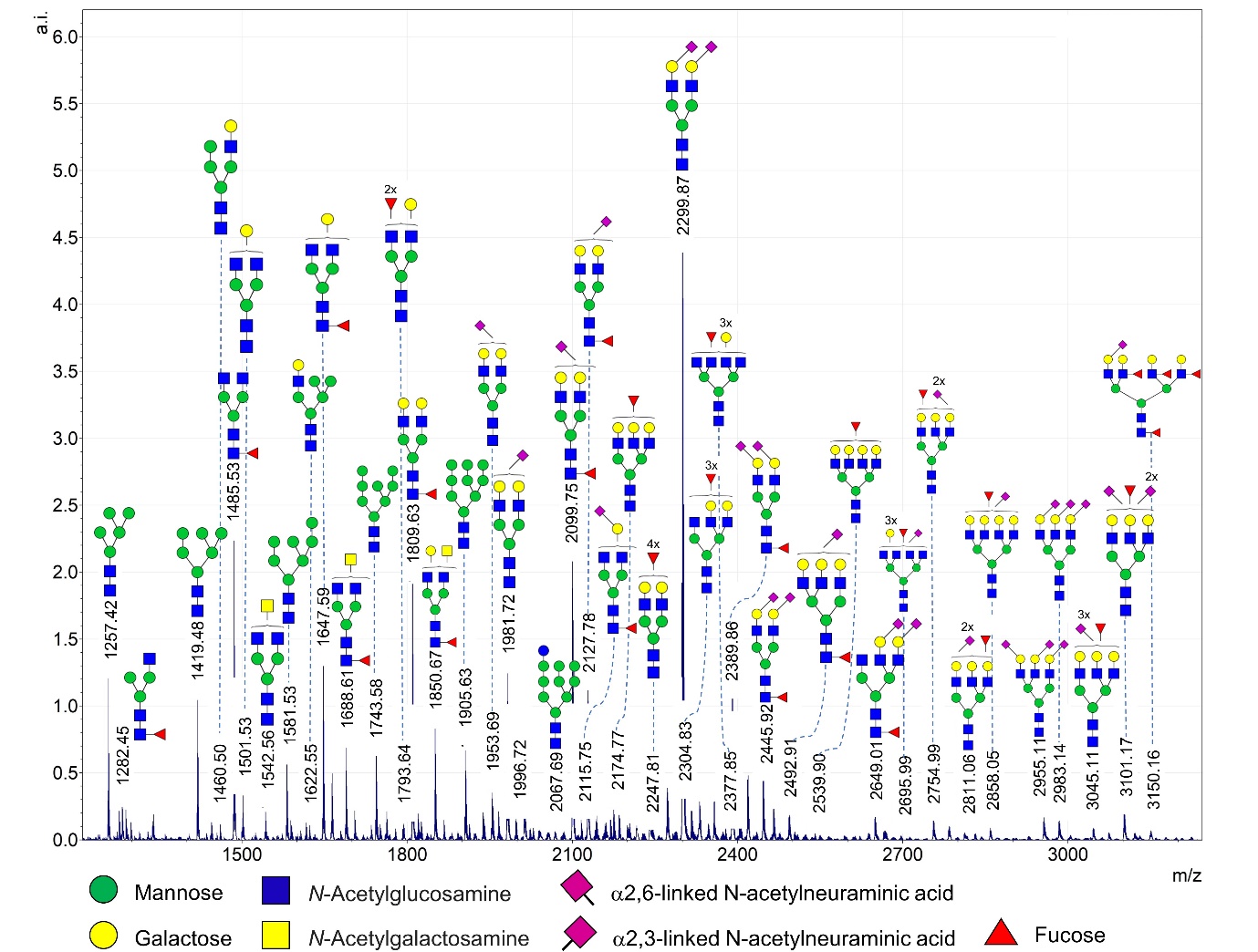


**Supplementary Figure S1: MALDI-TOF-MS** **N-glycome spectra of stage II CRC.** Overall average mass spectrum, representing the detected N-glycome of the full stage II CRC patient cohort. N-glycans were detected as sodium adduct ions [M+Na]^+^. Major glycan peaks were annotated and represent compositions based on MS/MS data and mass matching to theoretical composition, following N-glycan biosynthesis.


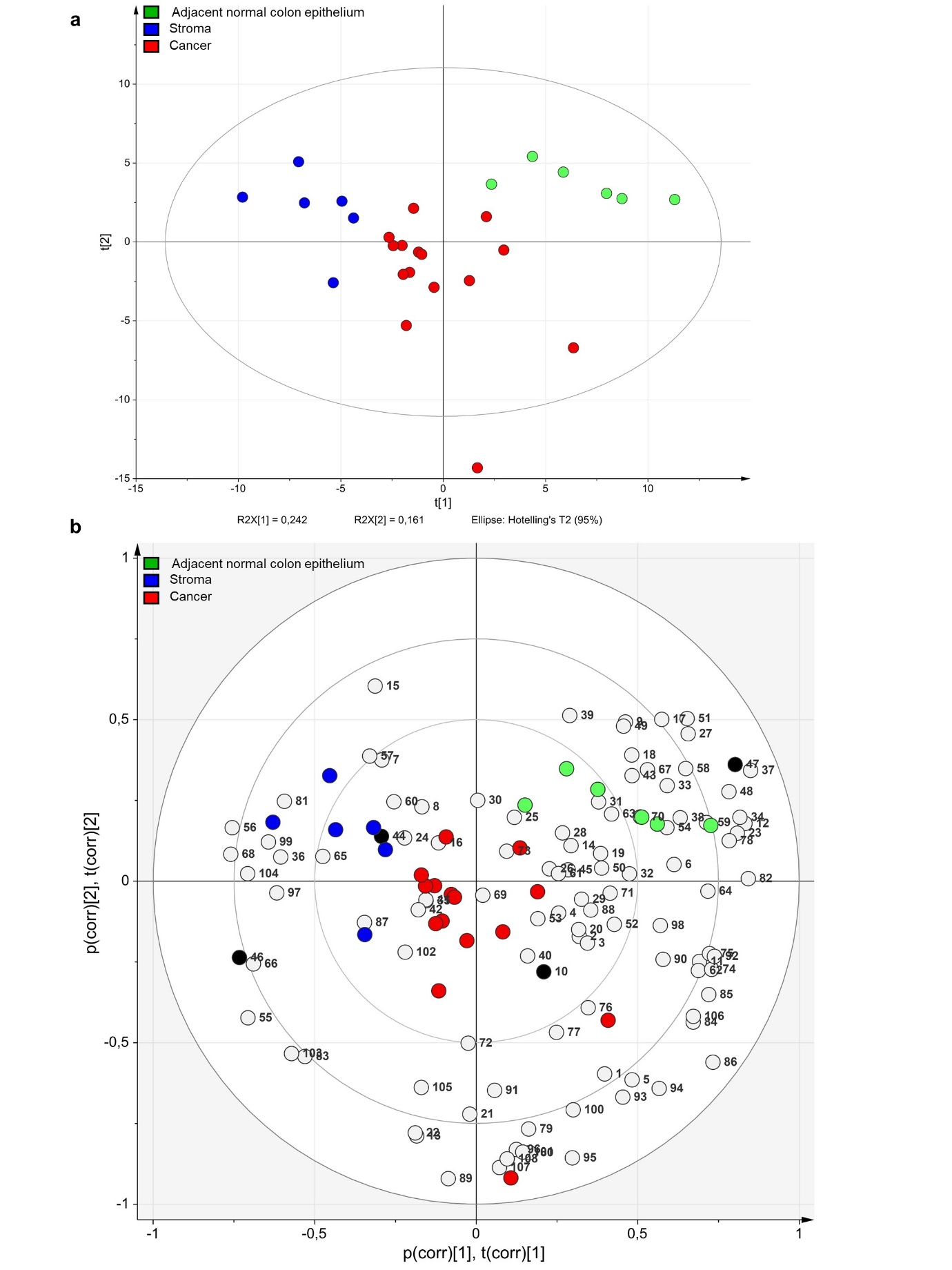


**Supplementary Figure S2: Separation of CRC morphologies based on their N-glycome.** **(a)** Scatter plot of the PCA 1 and 2 of the direct N-glycan trait**. (b)** Corresponding biplot with the co-chart score and loading together of the direct N-glycans for the morphologies normal epithelium, cancer and stroma distant. The spatial distributions of the four glycans highlighted in black can be found in Fig. 1. The code number of the glycan can be found in the supplementary Data S1.


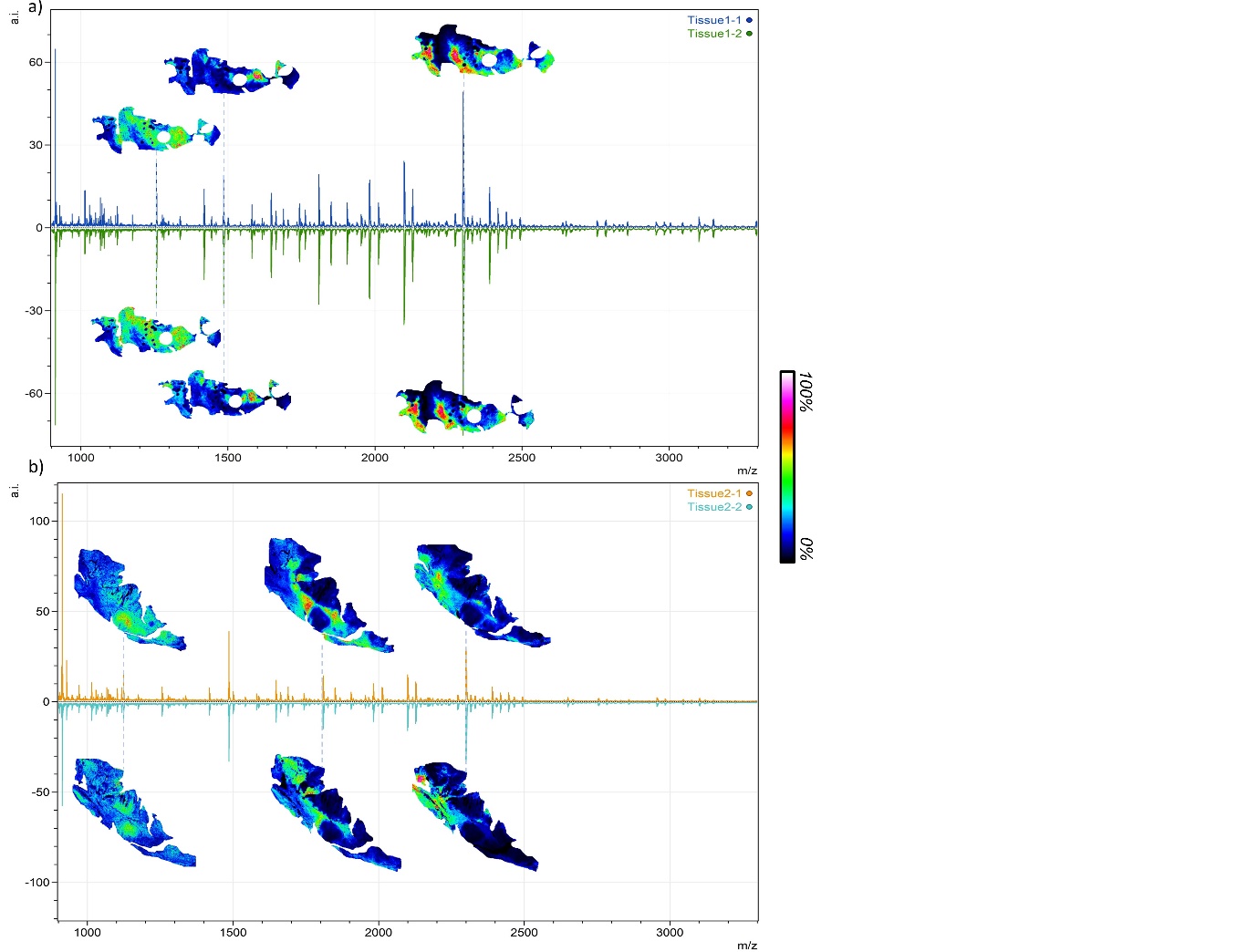


**Supplementary Figure S3: Technical replicates.** Overall average mass spectra of the technical replicates. a) Tissue1-1 and its replicate tissue1-2 and b) Tissue2-1 and its replicate tissue2-2. All spectra are TIC normalized and the distribution of 3 of the most abundant N-glycans have been added.
